# Supplementary material for: Fall Armyworm-Induced Secondary Metabolites in Sorghum Defend Against Its Attack
Source: Insects. 2025 Feb 17;16(2):218. doi: 10.3390/insects16020218 (PMC11856983; doi:10.3390/insects16020218)
Supplement: Supplementary file 1 [file insects-16-00218-s001.zip › insects-3447557-supplementary.pdf]

*Supplementary materials for*

# Fall armyworm-induced secondary metabolites in sorghum defend against its attack

Contents:

**Figure S1**. Effect of three secondary compounds on the relative growth rate of *Spodoptera frugiperda* larvae.

**Table S1.** Detailed classification of differential secondary metabolites in maize and sorghum groups.

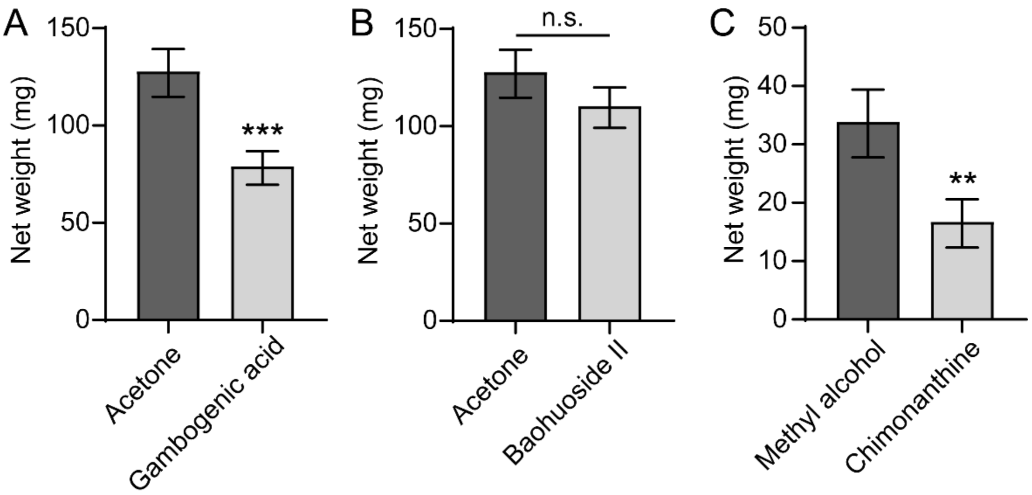


**Figure S1**. Effect of three secondary compounds on the relative growth rate of *Spodoptera frugiperda* larvae. The relative growth rate of $2^{nd}$ instar larvae of *S. frugiperda* to gambogenic acid (**A**), baohuoside II (**B**), and chimonanthine (**C**) from day 3 to day 5. Data are presented as mean ± SEM. ** $P < 0.01$, *** $P < 0.001$; n.s. indicates no significant difference between control and treatment ($p > 0.05$).

**Table S1.** Detailed classification of differential secondary metabolites in maize and sorghum groups.

| Number | Type | Name | Number | Type | Name |
|---|---|---|---|---|---|
| 1 | Monoterpenoid | Paederoside | 39 | Benzoic acid | 6-Paradol |
| 2 | Monoterpenoid | Valepotriate | 40 | Benzoic acid | Erianin |
| 3 | Monoterpenoid | Harpagide | 41 | Benzoic acid | Protocatechuic acid |
| 4 | Monoterpenoid | Oleuropein | 42 | Benzoic acid | TBHQ |
| 5 | Monoterpenoid | Rehmannioside D | 43 | Benzoic acid | Olivetol |
| 6 | Monoterpenoid | Benzoylpaeoniflorin | 44 | Hydrocinnamic acid | 4-Coumaric acid |
| 7 | Sesquiterpene | Artemisinin | 45 | Hydrocinnamic acid | 3-Methoxycinnamic acid |
| 8 | Sesquiterpene | Eupalinolide A | 46 | Hydrocinnamic acid | Martynoside |
| 9 | Sesquiterpene | Artemether | 47 | Hydrocinnamic acid | Lupulin A |
| 10 | Sesquiterpene | Curcumol | 48 | Coumarin | Isopimpinellin |
| 11 | Sesquiterpene | Sclareolide | 49 | Coumarin | 7-Hydroxy-4-methyl-8-nitrocoumarin |
| 12 | Sesquiterpene | Dihydroartemisinic acid | 50 | Coumarin | Curcumin |
| 13 | Sesquiterpene | Abscisic acid | 51 | Other | Rhapontin |
| 14 | Sesquiterpene | Alantolactone | 52 | Flavone | Wogonoside |
| 15 | Diterpene | Diosbulbin B | 53 | Flavone | Plantagoside |
| 16 | Diterpene | Danshenol C | 54 | Flavone | Vitexin-2-*O*-rhamnoside |
| 17 | Diterpene | Pseudolaric acid B | 55 | Dihydroflavone | Naringenin |
| 18 | Diterpene | Dehydroeffusol | 56 | Dihydroflavone | Narirutin |
| 19 | Diterpene | Kirenol | 57 | Flavonol | Laricitrin |
| 20 | Diterpene | Ingenol | 58 | Flavonol | Camelliaside A |
| 21 | Diterpene | 10-Deacetylbaccatin III | 59 | Flavonol | Baohuoside I |
| 22 | Diterpene | Euphorbia factor L1 | 60 | Flavonol | Avicularin |
| 23 | Triterpene | Obacunone | 61 | Flavonol | Baohuoside II |

| | | | | | |
|---|---|---|---|---|---|
| 24 | Triterpene | Ouabain | 62 | Isoflavone | Corylin |
| 25 | Triterpene | Kaji-ichigoside F1 | 63 | Isoflavone | Irisflorentin |
| 26 | Triterpene | Cucurbitacin I | 64 | Chalcone | Naringenin chalcone |
| 27 | Triterpene | Polygalic acid | 65 | Dihydrochalcone | Naringin dihydrochalcone |
| 28 | Triterpene | Trillin | 66 | Dihydrochalcone | Neohesperidin dihydrochalcone |
| 29 | Triterpene | Curcurbitacin IIA | 67 | Homoisoflavone | Methylophiopogonone A |
| 30 | Triterpene | Ziyuglycoside I | 68 | Isoflavane | Isomucronulatol |
| 31 | Triterpene | Ganoderic acid C6 | 69 | Other | Gambogenic acid |
| 32 | Triterpene | Cucurbitacin E | 70 | Indolizidine | Securinine |
| 33 | Triterpene | Notoginsenoside R1 | 71 | Pyrroloindole | Chimonanthine |
| 34 | Triterpene | Celastrol | 72 | Purine | Theophylline |
| 35 | Triterpene | Pristimerin | 73 | Quinoline | 4-Hydroxyquinazoline |
| 36 | Benzoic acid | Syringic acid | 74 | Quinoline | Pilocarpine |
| 37 | Benzoic acid | 2,4-Dihydroxybenzoic acid | 75 | Quinoline | Isoquinoline |
| 38 | Benzoic acid | 2,5-Dimethylphenol | 76 | Phenethylamine | Hordatine B |
